# Neural stem/progenitor cell properties of glial cells in the adult mouse auditory nerve

**DOI:** 10.1038/srep13383

**Published:** 2015-08-26

**Authors:** Hainan Lang, Yazhi Xing, LaShardai N. Brown, Devadoss J. Samuvel, Clarisse H. Panganiban, Luke T. Havens, Sundaravadivel Balasubramanian, Michael Wegner, Edward L. Krug, Jeremy L. Barth

**Affiliations:** 1 Department of Pathology and Laboratory Medicine, Medical University of South Carolina, Charleston, South Carolina 29425, United States; 2 Department of Regenerative Medicine and Cell Biology, Medical University of South Carolina, Charleston, South Carolina 29425, United States; 3 Department of Radiation Oncology, Medical University of South Carolina, Charleston, South Carolina 29425, United States; 4 Institute of Biochemistry, Friedrich-Alexander-University Erlangen-Nürnberg, Erlangen 91054, Germany

## Abstract

The auditory nerve is the primary conveyor of hearing information from sensory hair cells to the brain. It has been believed that loss of the auditory nerve is irreversible in the adult mammalian ear, resulting in sensorineural hearing loss. We examined the regenerative potential of the auditory nerve in a mouse model of auditory neuropathy. Following neuronal degeneration, quiescent glial cells converted to an activated state showing a decrease in nuclear chromatin condensation, altered histone deacetylase expression and up-regulation of numerous genes associated with neurogenesis or development. Neurosphere formation assays showed that adult auditory nerves contain neural stem/progenitor cells (NSPs) that were within a Sox2-positive glial population. Production of neurospheres from auditory nerve cells was stimulated by acute neuronal injury and hypoxic conditioning. These results demonstrate that a subset of glial cells in the adult auditory nerve exhibit several characteristics of NSPs and are therefore potential targets for promoting auditory nerve regeneration.

Degeneration of spiral ganglion neurons (SGNs) and their processes commonly occurs with aging, genetic mutations, and cochlear injuries caused by noise or ototoxic drug exposure. Studies of human temporal bones have shown that one of the most common pathological changes observed in age-related hearing loss is the degeneration of SGNs^1^,^2^. Damage to the auditory nerve and SGNs may occur not only secondarily to sensory hair cell loss, but also primarily in response to acoustic overexposure^3^. It has been believed that loss of spiral ganglion neurons and auditory nerve fibers are irreversible in the adult ear without external intervention, resulting in permanent sensorineural hearing loss (SNHL). The transplantation of neural stem/progenitor cells (NSPs) to facilitate the regeneration of neural tissues offers a promising therapeutic strategy for treating a variety of neurodegenerative disorders, including SNHL^4^,^5^,^6^,^7^. However, evidence from studies of various animal models of neurodegenerative disease indicates that the temporal window for the successful transplantation of NSPs after nerve injury is very short and that long-term survival and integration of NSPs in the chronically injured host environment is limited^8^,^9^,^10^. Previous studies showed that proliferative NSPs can be isolated from the auditory nerve of the perinatal cochlea^11^,^12^. It is essential to determine whether the self-renewing capability is still conserved in the endogenous cells of the adult auditory nerve.

NSPs have been characterized in several locations in the adult nervous system, including the subgranular zone (SGZ) of the dentate gyrus, the subventricular zone (SVZ) of the lateral ventricle, and the spinal cord after injury^13^,^14^. Brain injury and certain neurodegenerative disorders stimulate the proliferation of NSPs located in the SGZ and SVZ of the adult brain, and the resulting proliferative neural cells migrate into damaged brain regions. Interestingly, recent studies have demonstrated that the majority of these NSPs have characteristics typical of glial cells^15^. For example, NSPs in the SVZ and SGZ express several molecular markers associated with prototypic astrocytes, including Nestin, Gfap, S100β, the aldehyde dehydrogenase family, glulatamate transporters, and excitatory amino acid transporter 1 and 2^16^,^17^,^18^. Various phenotypical states of the astrocyte were identified during postnatal myelination and demyelination following homeostatic disturbance and injury in adult brain^19^,^20^. During these events, reactive astrocytes play an important role in promoting and modulating proper myelination or remyelination. Although it has been believed that severe adult astrocyte reactivity (or anisomorphic astrogliosis) has a significant negative impact on axonal regeneration, recent evidence suggests that astrocytes can act as stem/progenitor cells to promote adult nerve regeneration^18^,^21^. In our previous study, increases in Sox2^+^ cell number and glial proliferation were observed in the auditory nerve of the adult mouse cochlea shortly after ouabain exposure^22^. In the present study, we report characterization of the cellular and molecular alterations occurring in ouabain-treated ears and examined the regenerative capability of adult auditory nerves in response to SGN death with a focus on glial cells.

## Results

### Changes in cellular differentiation state of mature glial cells in the auditory nerve following ouabain injury

**Figure 1:**
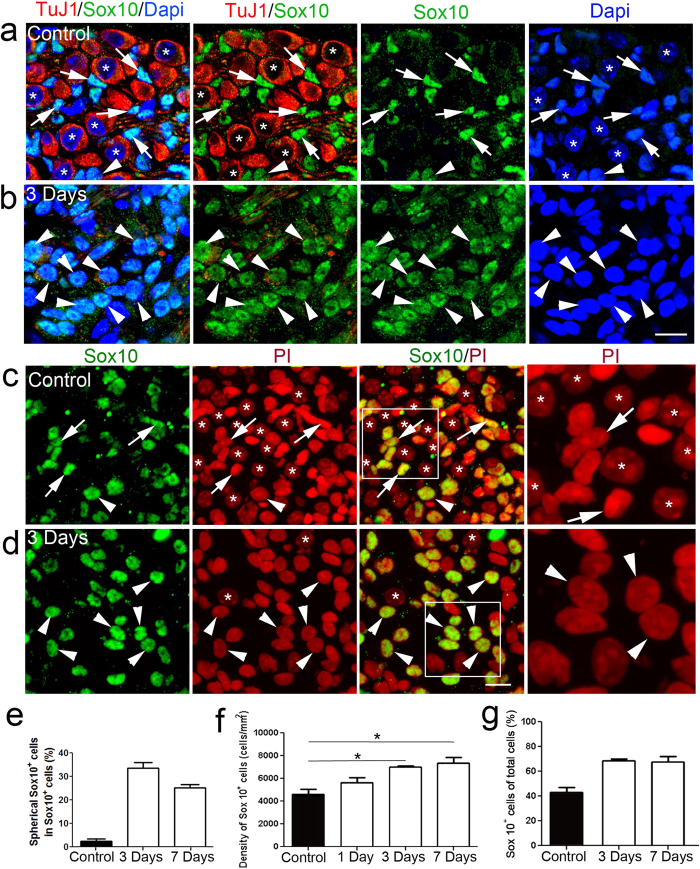
Cellular changes of Sox10^+^ glial cells in the injured auditory nerve. (**a**–**d**) Acute auditory nerve injury induces an increase in Sox10^+^ glial cells (green) with rounded nuclei. Spiral ganglion neurons were labeled with β Tubulin III (TuJ1, red; (**a**,**b**)). Nuclei were counterstained with Dapi (blue; (**a**,**b**)) and with propidium iodide (PI, red; (**c**,**d**)). Asterisks indicate Sox10^−^ neurons with a spherical nucleus. In the spiral ganglion of a control adult mouse, a large portion of Sox10^+^ glial nuclei (arrows) had a typical elongated spindle shape. Few Sox10^+^ cells had a spherical nucleus (arrowhead). In contrast, in the injured spiral ganglion of mice 3 days after ouabain exposure, most Sox10^+^ glial nuclei (arrowheads) had a spherical shape (**b,d**). The right panels in (**c**,**d**) are the enlarged images of boxed areas in the composite panels. (**e**) Quantitative analysis of spherical Sox10^+^ cells in control (n = 4), and 3 (n = 10) and 7 (n = 6) days after ouabain exposure. The spherical cells were identified as having nuclei with a long dimension/short dimension ratio of less than 1.5. (**f**) Injured auditory nerves show a significant increase in Sox10^+^ cells at 3 and 7 days after ouabain exposure. Mean density of Sox10^+^ cells ± S.E.M was obtained from control mice (n = 13) and treated mice at 1 (n = 5), 3 (n = 4) and 7 (n = 5) days after ouabain exposure. Asterisks indicate statistically significant differences between control and treated groups (ANOVA, *p* < 0.05). (**g**) Percenta**g**es of Sox10^+^ cells in the control and injured auditory nerves. Scale bars, 10 μm in (**b**) (applies to (**a**,**b**)); 10 μm in (**d**) (applies to the three left panels in (**c**,**d**)).

Ouabain treatment of adult rodent cochleas is a well-established model of selective type I SGN degeneration^22^,^23^. It has been shown that the Sox10 transcription factor is highly expressed in both mature and undifferentiated glial cells^24^,^25^. Here, we examined the consequences on Sox10^+^ glial cells in auditory nerves of ouabain-treated mouse cochleas. In adult control mice, the nuclei of Sox10^+^ glial cells appeared spindle-shaped and small compared to the rounded nuclei of SGNs (Fig. 1a,c,e). However, in ouabain-treated cochleas, nuclei of Sox10^+^ glia were significantly altered, appearing enlarged and having a rounded shape resembling nuclei of SGNs, which were labeled with neuronal marker, TuJ1 (Fig. 1b,d,e). These changes were seen in a large fraction of Sox10^+^ glial cells in both Rosenthal’s canal and the osseous spiral lamina portions of the auditory nerves at 3 and 7 days after ouabain exposure (Fig. 1e). Cell counts indicated that the density of Sox10^+^ glial cells increased significantly in ouabain-treated auditory nerves at 3 and 7 days after ouabain exposure (ANOVA, *p* < 0.05; Fig. 1f), increasing from approximately 40% in adult controls to 70% in injured samples (Fig. 1g).

**Figure 2:**
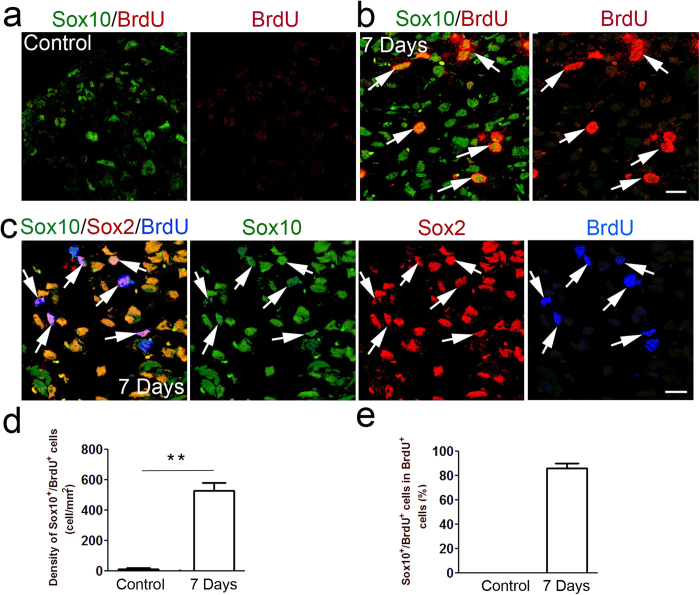
Increased proliferative Sox10^+^ glial cells in the injured auditory nerve. (**a**–**e**) A significant increase in BrdU^+^/Sox10^+^ cells (arrows) was seen 7 days after ouabain exposure. 6/8 BrdU^+^/Sox10^+^ cells in (**c**) also expressed the Sox2 transcription factor (arrows). Mean density of BrdU^+^/Sox10^+^ cells ± S.E.M was obtained from control mice (n = 4) and treated mice at 7 days (n = 6) after ouabain exposure. The percentage of BrdU^+^/Sox10^+^ cells in total BrdU^+^ cells at 7 days after ouabain exposure was plotted in (**e**). Scale bars, 10 μm in (**a**) (applies to (**a**,**c**)); 10 μm in (**c**).

Numerous Sox10^+^/BrdU^+^ cells were seen in ouabain-treated auditory nerves, whereas only few Sox10^+^/BrdU^+^ cells were found in controls (Fig. 2), suggesting that the increase in Sox10^+^ glia resulted from cell proliferation. In addition, most of the proliferative Sox10^+^ glial cells in treated nerves also expressed Sox2 (Fig. 2c). These data are supported by our previous observations that ouabain exposure causes a significant increase in BrdU^+^ cells in the injured auditory nerve and that a majority (around 80%) of BrdU^+^ cells are Sox2^+^ glial cells at 7 days after ouabain exposure^22^.

**Figure 3:**
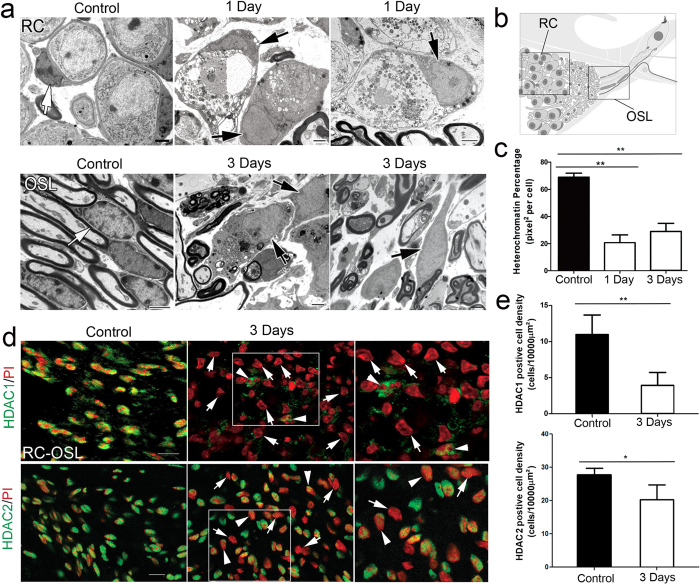
Changes of euchromatin/heterochromatin ratios and evidence of histone deacetylation in the glial cells of the injured auditory nerve. (**a**) Alterations of nuclear ultrastructure in glial cells within Rosenthal’s canal (RC) and the osseous spiral lamina (OSL). In the control ears, glial cells (white arrows), which wrap around the perikarya of type I neurons and their processes, had nuclei with prominent electron-dense heterochromatin (patches of dark coloration). 1–3 days after ouabain exposure, glial cell nuclei exhibit less heterochromatin but more of the lightly colored euchromatin (black arrows). (**b**) A schematic diagram showing the area of the OSL and RC in the peripheral mouse auditory nerve. (**c**) Quantitative analysis of nuclear subdomains showed a significant decrease of heterochromatin percentage in the glial cell nuclei of injured auditory nerves (n = 6 per group) as compared to controls (n = 11 per group; Student’s unpaired t-test; *p < 0.05; **p < 0.01). (**d**) Injured auditory nerves showed a decline in HDAC 1 (top row) and HDAC2 (bottom row) immunoreactivity. The majority of glial cells in control auditory nerve were positive for HDACs (green), whereas numerous glial cells within an injured auditory nerve were negative for HDAC (arrows) or showed a reduced level of HDAC (arrow heads) in the cytoplasm. Right panels show the enlarged images of the boxed areas. (**e**) Quantitative analysis of HDAC expr**e**ssed cells were conducted at 3 days after ouabain exposure (n = 3–4 per group; Student’s unpaired t-test; *p < 0.05). Note that that glial cells showed only a modest reduction in the amount of HDAC2^+^ cells but a larger reduction in HDAC1^+^ cells after ouabain exposure for 3 days. Scale bars, 2 μm in (**a**); 10 μm in (**d**).

The observation of glial nuclear changes by light microscopy led us to perform further ultra-structural characterization. In adult control auditory nerves, the nuclei of glial cells contained dense patches of heterochromatin but revealed little euchromatin (Fig. 3a). In contrast, in ouabain-injured ears the glial cell nuclei showed less heterochromatin, but abundant euchromatin, suggesting increased gene transcription. Quantitative analysis of the amount of heterochromatin (percentage of total nuclear area) revealed a significant reduction of heterochromatin in glial cell nuclei at 1 and 3 days after ouabain treatment (Fig. 3c), indicating that glial cells in the adult auditory nerve change from an inactive to an activated state shortly after ouabain injury.

Reduction in histone deacetylase (HDAC) activity can limit histone hypoacetylation, leading to transcriptional activation^26^. We therefore examined the production of HDACs in the auditory nerve as a consequence of injury. In control mice, the majority of spindle-shaped glial nuclei stained positively with both HDAC1 and HDAC2 antibodies (Fig. 3d). However, 3 days after ouabain treatment, there was a significant reduction in HDAC1 and HDAC2 immunoreactivity in glial cells (Fig. 3e). Interestingly, nerve injury caused approximately a two thirds reduction in HDAC1^+^ cells but only a one third reduction in HDAC2^+^ cells (Fig. 3e), suggesting distinct roles of these two HDACs in regulating chromatin remodeling and glial phenotypical alteration in the injured adult auditory nerve.

### Recapitulation of neurogenic gene expression patterns in adult auditory nerve following ouabain injury

**Figure 4:**
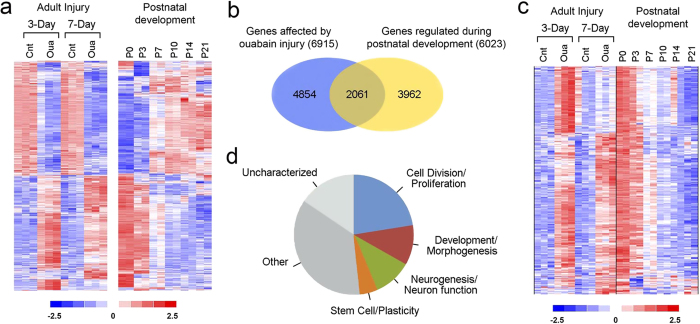
Gene expression profiles in injure**d** auditory nerve recapitulate aspects of auditory nerve postnatal development. (**a**) Expression profiles of genes differentially expressed in adult auditory nerves at 3 days and 7 days after ouabain injury was shown in the left panel. 6915 genes were identified by the criteria of fold changes >1.5 and *p* < 0.05 (Student’s unpaired t-test) at either 3 days or 7 days after ouabain exposure. Expression profiles of genes differentially expressed in postnatal auditory nerves are present in the right panel. 6023 genes were identified as significantly different by the criteria of fold changes >1.5 and *p* < 0.05 (Student’s paired t-test) at either P3, P7 or P10 compared to P0. Colorimetric scaling (Z-standardization) is indicated at the bottom. (**b**) A Venn diagram showed that many of the genes differentially expressed following ouabain exposure are also differentially expressed during postnatal development (2061 of 6915). (**c**) A heat map displayed expression profiles of 636 genes that were up-regulated in adult auditory nerves by injury and also highly expressed at the onset of auditory nerve development (P0) but down-regulated during postnatal development. (**d**) Functional categorization of the 636 genes depicted in panel (**c**).

Based on the changes observed in response to ouabain injury, we hypothesized that the injury response included a regenerative component that involved glial cells. To explore molecular aspects of regenerative potential following injury, microarray transcriptional profiling was performed on auditory nerve preparations from ouabain-treated animals either 3 days or 7 days after treatment. As a reference for these studies, profiling was also performed on auditory-nerve-enriched samples from mice at postnatal days (P) 0, 3, 7, 10, 14, and 21, reasoning that regeneration may resemble aspects of normal auditory nerve development. Analysis of the injury data revealed that 6915 genes were differentially expressed at either 3 days or 7 days following injury (Fig. 4a; Supplementary Table 1). Analysis of the postnatal development data identified 6023 genes that were differentially expressed at P3, P7, or P10 compared to P0 (Fig. 4a; Supplementary Table 2).

Evaluation of both the injury and the developmental data sets together revealed that 2061 genes were altered in both models (Fig. 4b). We predicted that a subset of the genes activated by injury would be genes that are normally highly active during early postnatal development. Thus, the activation of these genes by injury would represent a neurogenic response that resembled the early stages of postnatal auditory nerve development. Analysis of the microarray data detected 636 genes that matched the predicted profile of up-regulation following ouabain injury and developmental expression that was highest at the earliest postnatal stage, P0 (Fig. 4c). Analysis of gene ontology terms associated with the 636 genes showed that approximately half were linked to cellular responses including cell division/proliferation, development/morphogenesis, neurogenesis/neuron function, and stem cell/plasticity (Fig. 4d; Supplementary Table 3). An additional analysis involving a previously published microarray data set for vestibular ganglion development^27^ confirmed that a large portion of the 636 genes were also highly expressed in the vestibular ganglion at embryonic day 16 and P0 (Supplementary Fig. 1; Supplementary Table 4).

**Figure 5:**
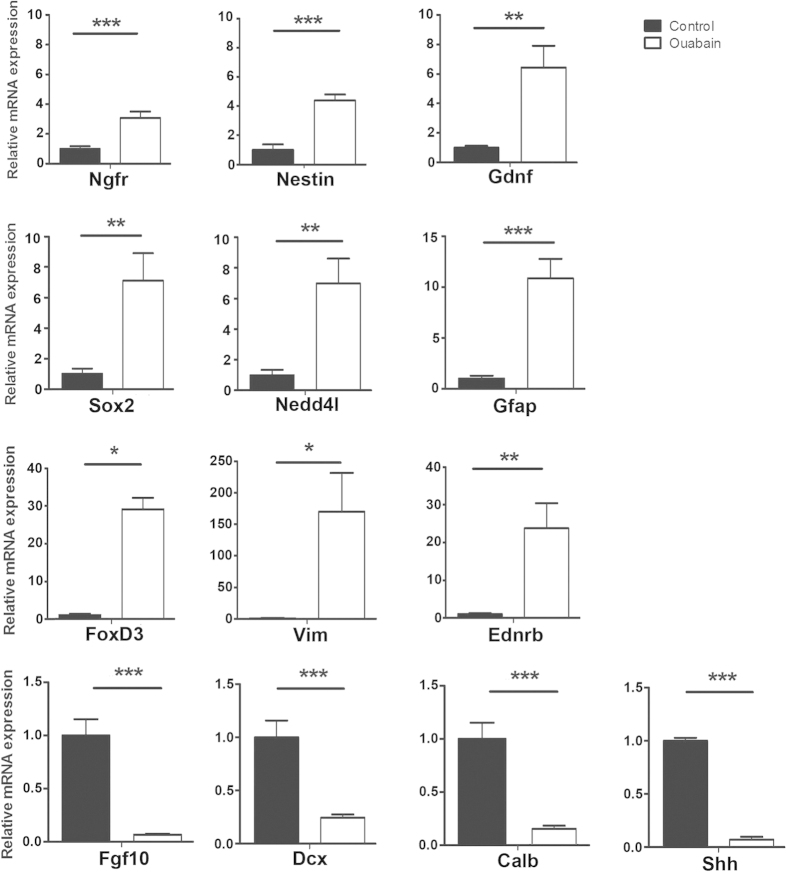
Validation of differential gene expression in ouabain-injured auditory nerve. Quantitative RT-PCR analysis of injured auditory nerves 3 days after ouabain exposure validated differential expression of 13 genes identified by microarray experimentation. 18s was used as internal control. Data are presented as fold change relative to controls ± standard error (n = 3 assays per group; 2 auditory nerves per assay). Student’s paired t-test; a value of *p* < 0.05 (*) was considered to be statistically significant. **p < 0.01; ***p < 0.001.

Differential expression of 13 genes identified by microarray analysis was confirmed by quantitative RT-PCR. These genes were chosen based on their well-characterized regulation of neural development and/or NSP proliferation and differentiation^28^,^29^,^30^,^31^. Among the selected set, 9 up-regulated genes (Ngfr, Nestin, Sox2, Nedd4l, Gfap, Ednrb, Gdnf, FoxD3 and Vim), and 4 down-regulated genes (Fgf10, Dcx, Calb and Shh) were confirmed to be significantly affected in ouabain-treated auditory nerves (Fig. 5). Some of the up-regulated genes such as Sox2, Nestin, Vim, Gfap, and Ngfr (p75) are widely used as NSP markers^32^,^33^,^34^.

To investigate transcriptional mechanisms that might contribute to the neurogenic gene expression response occurring in injured auditory nerve, we performed an analysis of promoter sequences of the 398 genes that showed a profile of up-regulation at both 3 and 7 days after ouabain injury and a profile of high expression at P0, but down-regulation with age during postnatal auditory nerve development. Interestingly, one of the transcriptional regulatory elements (TREs) significantly enriched within this group of gene promoters was the binding site for FoxD3 (Z = 10.462; Table 1), a member of the forkhead box family of transcription factors. FoxD3 was initially characterized in neural crest-derived stem cells (NCSCs) and embryonic stem cells, and it has been shown to be critical in NCSCs for maintaining neural differentiation potential and repressing mesenchymal lineage fates^35^,^36^. Putative FoxD3 binding sites were present in promoters of 160 of the 368 genes analyzed.

**Figure 6:**
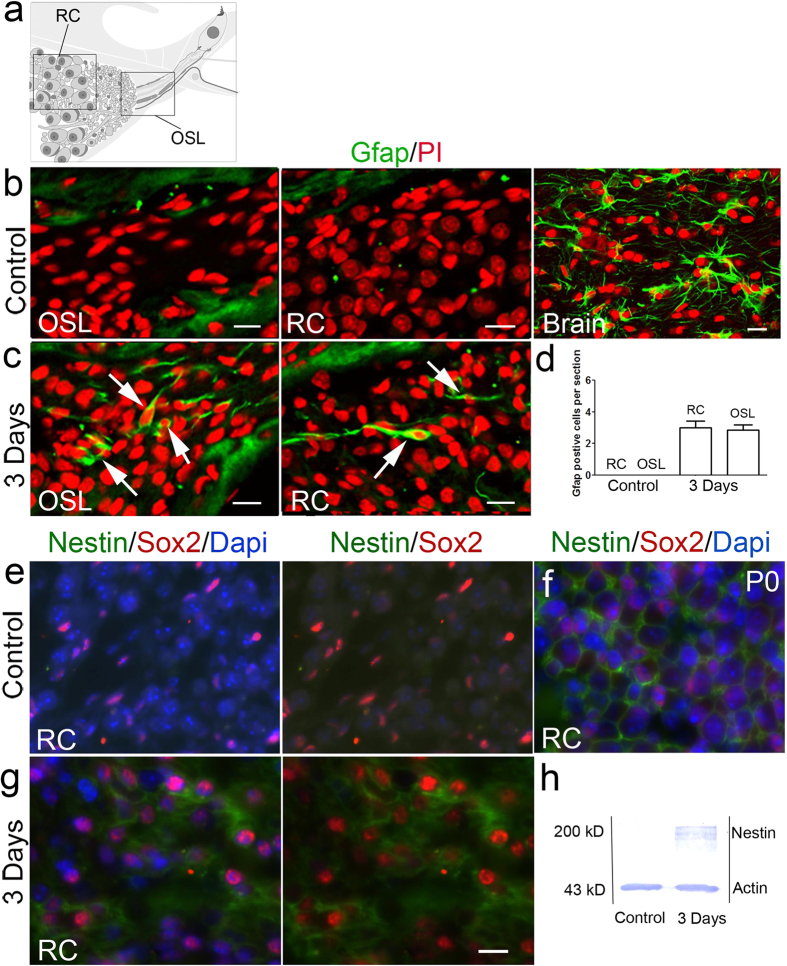
Gfap^+^ glial cells appear in the peripheral portion of mouse injured auditory nerves. (**a**) A schematic diagram depicts the OSL and RC regions in the peripheral mouse auditory nerve. (**b**) No Gfap^+^ cells were seen in the auditory nerve periphery in RC and OSL of normal adult mice. Detection of Gfap^+^ oligodendrocytes present in the brain of the same mouse was performed as a positive control. Gfap^+^ oligodendrocytes were also present in the adult mouse brain. (**c**,**d**) Gfap^+^ glial cells (arrows, green) were present in the auditory nerve periphery in RC and OSL 3 days after ouabain treatment. Gfap^+^ cells were counted in sections of the OSL (n = 7) and RC (n = 5) for controls and ouabain exposed samples. (**e**) No Nestin immunoreactivity was detected in the auditory nerve of a normal adult mouse. (**f**) Cytoplasmic Nestin (green) immunoreactivity was detected in the auditory nerve of a P0 mouse. (**g**) Nestin immunoreactivity was present in the cytoplasm of Sox2^+^ glial cells in the auditory nerve 3 days after ouabain exposure. (**h**) Western blot analysis detected an increase in Nestin protein (200 kD) in auditory nerves (n = 6) 3 day after ouabain exposure. β-actin blotting was used to verify equal protein loading. Scale bars, 10 μm in (**b**,**c**); 10 μm in (**g**) (applies to (**e**–**g**)).

Glial fibrillary acidic protein (Gfap), the major intermediate filament in astrocytes of the central nervous system, is important in modulating astrocyte motility. Gfap^+^ cells are thought to be the principal source of NSPs in postnatal and adult mouse forebrain^28^,^37^. The intermediate filament protein Nestin is highly expressed during neurogenesis and has been widely used as a ubiquitous marker for NSPs^38^. Our microarray and RT-PCR assays revealed that Gfap and Nestin mRNA was up-regulated in adult auditory nerves after ouabain exposure. To further investigate this response, we conducted immunostaining for these proteins on cochlear sections prepared from control and ouabain-treated mice. As shown in Fig. 6b, glial cells in the auditory nerve periphery (OSL and RC) were negative for Gfap staining. However, in the ouabain-treated mice, Gfap^+^ glial cells appeared in both OSL and RC (Fig. 6c,d). Thus acute SGN damage activated production of the potential NSP marker Gfap in a population of cells in the peripheral auditory nerve. Nestin protein expression was also up-regulated in the injured auditory nerves based on immunostaining as well as western blot analysis (Fig. 6e–g).

### Isolation and generation of neurospheres from adult auditory nerve

**Figure 7:**
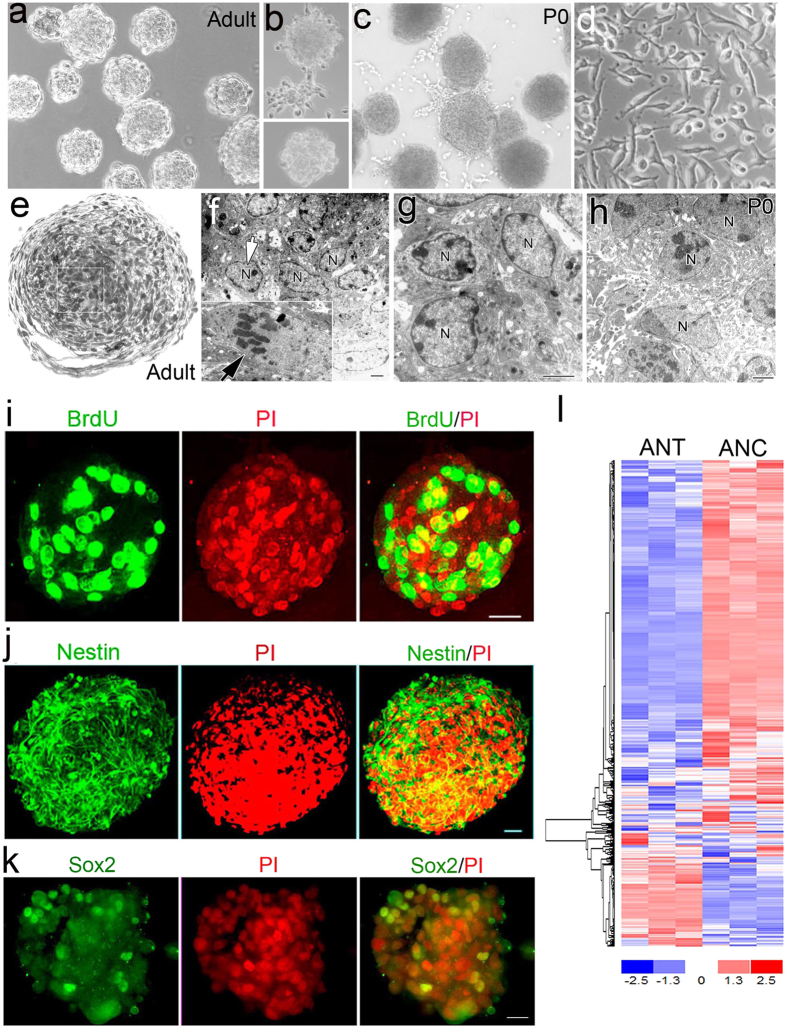
Neurospheres can be produced from adult auditory nerve cells and exhibit molecular characteristics of neural st**e**m/progenitor cells. (**a**) A DIC image showing typical morphology of neurospheres generated after 12 days in culture with NeuroCult^TM^ NSC proliferation supplement. (**b**) Second-passage neurospheres (upper panel) and third-passage neurospheres (lower panel) were morphologically similar to primary spheres. (**c**) Neurospheres generated from the auditory nerves of a newborn mouse. (**d**) Fibroblast cultures (L cell, ATCC^@^ CRL-2648^TM^) did not generate neurospheres. (**e**) Micrograph showing a cross-section of a neurosphere generated from adult mouse auditory nerve. The resin-embedded, semi-thin section was stained with toluidine blue. (**f**,**g**) Ultrastructural characterization of neurospheres from adult auditory nerves showing a diverse population of cells in varyin**g** states of differentiation. Images were taken from the boxed area of the neurosphere shown in (**e**). A mitotic nucleus is shown in an inset panel at the left bottom of (**f**). The majority of cells showing a high nucleus (N) to cytoplasm ratio. (**h**) Ultrastructural features of a neurosphere section from the auditory nerve of a P0 CBA mouse. (**i**,**j**) A majority of neurosphere-derived cells were positive for BrdU and Nestin. Nuclei were counterstained with PI. (**k**) A cross section of a single neurosphere showing that cells express Sox2, another NSC marker. Scale bars, 30 μm in (**a**–**c**). (**l**) Adult stemness genes were regulated and predominantly activated in auditory nerve-derived neurosphere cultures (ANC) compared to adult auditory nerve tissue (ANT). Significant difference was defined as *p* < 0.05, Student’s unpaired t-test. Scale bars, 2 μm in (**f**–**h**); 25 μm in (**j**,**k**).

The neurosphere-formation assay is widely used to detect stem/progenitor cells and assess self-renewal and differentiation in cochlear tissues^11^,^39^,^40^. We used a modified neurosphere formation protocol^37^,^41^ to examine the consequences of acute injury. Clonal density cells prepared from auditory nerves of adult mice were able to form neurospheres (Fig. 7a), as were cells from the auditory nerves of newborn mice (Fig. 7c). Neurospheres prepared from auditory nerve could be enzymatically dissociated and the isolated cells again subjected to the culture protocol, which resulted in the formation of new neurospheres. Neurosphere cells generated from adult auditory nerves were able to generate neurospheres for at least three passages (Fig. 7b), whereas neurosphere cells from newborn mice yielded neurospheres for more than seven passages. As a negative control, fibroblast cells isolated from mouse subcutaneous connective tissue (L cell line, ATCC^@^ CRL-2648^TM^) were subjected to the formation protocol, but no sphere-like clusters were produced (Fig. 7d).

Ultrastructural characterization of adult auditory nerve neurospheres revealed morphologically heterogeneous cells in varying states of differentiation, including presumed stem/progenitor cells (Fig. 7a,b,d,e). Neurosphere cells had a non-compact cytoarchitecture with multiple cellular processes and large intercellular spaces (Fig. 7f,g). Neurosphere cells showed cytoplasms with irregular contours and short or no expansions. Additionally neurosphere cells showed a high nuclear/cytoplasm ratio (Fig. 7f,g) which was also evident in neurospheres prepared from newborn auditory nerves (Fig. 7h). The majority of neurosphere cells had abundant rough endoplasmic reticulum, mitochondria, and microfilaments. Mitotic features were occasionally detected in the central areas of the spheres (Fig. 7f). No cells with typical ultrastructural features of mature neurons or glial cells were found in the neurosphere sections.

As shown in Fig. 7i, a large subset of neurosphere cells from adult mice was proliferative, based on a BrdU assay. The intermediate filament protein Nestin and the transcription factor Sox2 are two widely used cellular markers for NSPs^38^,^42^. In preparations of whole spheres or sphere sections, numerous cells were found to be Nestin^+^ and Sox2^+^ (Fig. 7j,k). To further explore stem-related characteristics of neurosphere cells, microarray analyses were conducted on neurospheres and auditory nerves from adult mice to examine expression of genes linked to stemness in adult cells^43^. Of the 506 adult stemness genes interrogated, a majority showed a significant difference (*p* < 0.05) in neurospheres compared to auditory nerves (346 genes; 68.7%; Fig. 7l; Supplementary Table 5). Furthermore, a disproportionate number of the significantly different genes showed up-regulation (282 of 346; 81.5%) versus down-regulation (64 of 346; 18.5%) in neurospheres. These findings support the interpretation that generation of neurospheres from adult auditory nerve involves established adult stem cell mechanisms.

**Figure 8:**
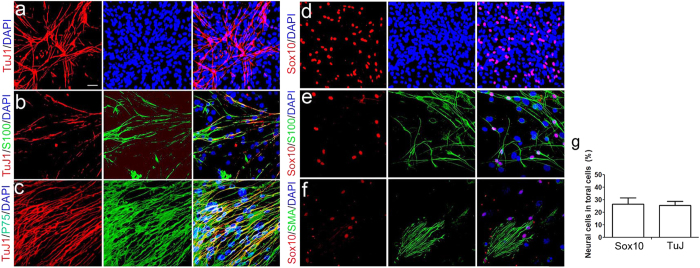
Neurosphere cells propagated under a neural differentiation condition are positive for neuronal and glial cell markers. (**a**–**f**) After 14 days of culture prepared from adult auditory nerves with NeuroCult^TM^ neural differentiation supplement, neurosphere cells express the neuronal marker, TuJ1, and several glial cell markers, including Sox10, P75, and S100. A small number of sphere-derived cells express SMA, a marker of myofibroblasts. (**g**) Quantitative analysis of Sox10^+^ (n = 6 individual cultures) and TuJ1^+^ cells (n = 8 individual cultures) in neurosphere cells prepared from adult auditory nerves. Scale bar, 45 μm in (**a**–**f**).

After growth in neural differentiation-promoting conditions for 10–12 days, neurosphere cells prepared from adult auditory nerves significantly changed their morphological features. A majority of cells changed from flat shapes with multipolar processes to spindle shapes with elongated bipolar processes (Fig. 8). Immunostaining revealed that a portion of neurosphere cells were stained positively for a panel of neural makers (Fig. 8a–f), including TuJ1 and MAP2 as neuronal markers, and P75, Sox10 and S100 as glial lineage markers. The TuJ1^+^ cells composed 25.6 ± 3.2% of the total cell population (n = 8) and Sox10^+^ cells were 26.6 ± 3.2% (n = 6) (Fig. 8g). A small proportion of the cells (less than 1%) were positive for the myofibroblast marker Smooth Muscle Actin (SMA).

### Isolation and generation of neurospheres from the glial cells of adult auditory nerve

**Figure 9:**
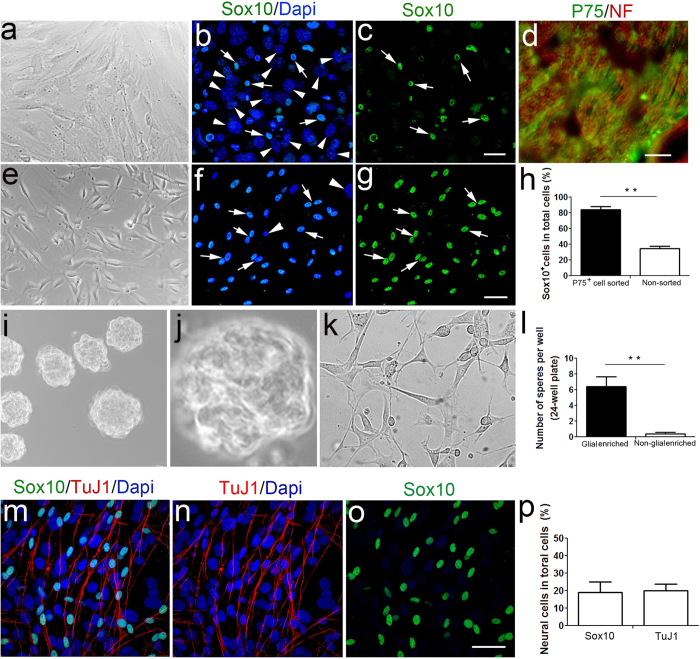
Neurospheres can be produced from glial cells isolated from adult auditory nerve. (**a**–**g**) Glial cell enrichment using immunomagnetic sorting with a glial cell surface maker P75. After cell sorting, a majority of the cells were identified as glial cells by their spindle-shaped morphology in differential interference contrast (DIC) view (**e**) and positive staining with Sox10 antibody (arrows) (**g**), whereas only a small portion of cells displayed the glial morphological characteristics (**a**) and stained positively with Sox10 antibody in non-sorted cell preparations (**c**). P75 immmunoreactivity (green) appeared on the surface of Schwann cells in the peripheral auditory nerve (**d**). Auditory nerve axons were labeled with neurofilament (NF, red) and cell nuclei were counterstained with Dapi (blue). White arrowheads indicate Sox10^−^ cells with large oval-shaped nuclei. (**i,j**) Neurospheres generated from glial enriched fractions. Image in (**j**) showed an enlarged view of a neurosphere. (**k**) A majority of the cells from the non-glial enriched fraction were attached the bottom of the culture dish and failed to generate neurosphere. (**l**) Comparison of the neurosphere formation capability between the glial enriched and non-glial enriched fractions. Mean number of neurospheres ± S.E.M was obtained from glial enriched fractions (n = 12) and non-glial enriched fractions (n = 4). Asterisks indicate statistically significant differences between the two groups (Student’s unpaired t-test, *p* < 0.05). (**m**–**p**) Neurosphere cells generated from glial enriched fractions stained positively with TuJ1 (red) and Sox10 (green) under neural differentiation conditions. Percentages of Sox10^+^ and TuJ1^+^ cells were tabulated from 4 individual cultures with glial enriched cell populations prepared from adult mouse auditory nerves. Scale bars, 25 μm in (**c**,**d**,**g**,**o**).

We sought to test the hypothesis that neurospheres produced from auditory nerve cultures originated from glial cells. A glial enriched population was obtained from adult mouse auditory nerve using an immunomagnetic cell sorting procedure adapted from that of Whitlon *et al.*^44^. Glial cells were separated based on cell surface expression of nerve growth factor receptor (P75) (Fig. 1d). After two rounds of immunomagnetic sorting, the P75^+^ cell fraction was more than 86% glia based on spindle-shaped morphology and Sox10 immunoreactivity (Fig. 9d–f), whereas the auditory nerve cell preparations without the immunomagnetic sorting contained less than 35% Sox10^+^ glial cells (Fig. 9a,b,f). The glia enriched fraction was then used in both the neurosphere formation and neural differentiation assays. As shown in Fig. 9l, the glial enriched fractions produced much more neurospheres than that of non-glial enriched fractions. Most cells from the non-glial enriched population remained attached to the culture dish and failed to generate neurospheres (Fig. 9k). Under neural differentiation conditions, neurosphere cells generated from the glial enriched fractions were able to differentiate into Sox10^+^ and TuJ1^+^ neural cells (Fig. 9m–o). Quantitative analysis of differentiated neurosphere cultures showed that Sox10^+^ and TuJ1^+^ cells comprised 19 ± 3% and 20 ± 3% of total cells, respectively.

### Sox2 expressing glial cells are neurosphere-forming cells

**Figure 10:**
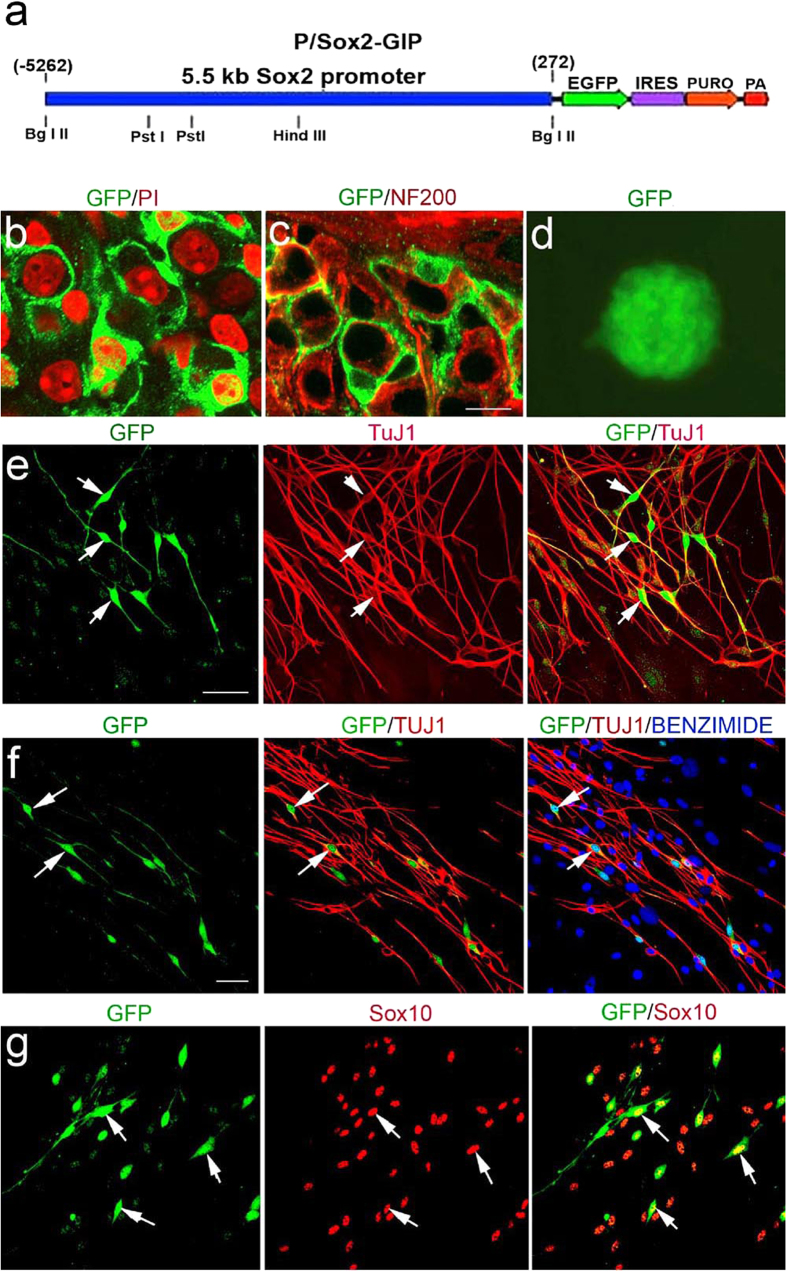
Sox2-expressing glial cells in adult auditory nerves have the properties of neural stem/progenitor cells. (**a**) Schematic structure of the Sox2-eGFP transgene. (**b**,**c**) Glial cells expressed GFP signifying Sox2 expression (arrows). PI^+^ neuron nuclei ((**b**), red) or NF200^+^ neuronal cell bodies ((**c**), red) were surrounded by GFP^+^ glial cells (green). (**d**) A large proportion of neurosphere cells expressed GFP (green). (**e**–**g**) Neurospheres propagated under neural differentiation regimen express neuronal and glial cell markers. Scale bars, 10 μm in (**b**,**c**); 45 μm in (**e**); 45 μm in (**f**,**g**).

Sox2 is a transcription factor that regulates the pluripotent state of stem cells and is also highly expressed in proliferative NSPs^42^,^45^. Analysis of a Sox2 eGFP transgenic mouse model^45^ revealed that a large portion of Schwann cells within Rosenthal’s canal and the osseous spiral lamina were Sox2^+^ (Fig. 10b). The glial nature of these GFP^+^ cells was determined by their morphological features and their spatial relationship with NF200^+^ spiral ganglion neurons (Fig. 10b,c). Auditory nerves isolated from adult Sox2 eGFP mice generated neurospheres (Fig. 10d) with the majority of neurosphere cells being GFP^+^. Culturing neurospheres in neural differentiation medium revealed that some differentiated neural lineage cells were GFP^+^. The neural lineage cells were identified based on positive staining with TuJ1 and Sox10 (Fig. 10e–g). Thus it appears that Sox2^+^ glial cells in the adult auditory nerve are a main source of the neurosphere forming cells.

### Enhanced neurosphere formation from auditory nerve by acute injury or hypoxic conditions

**Figure 11:**
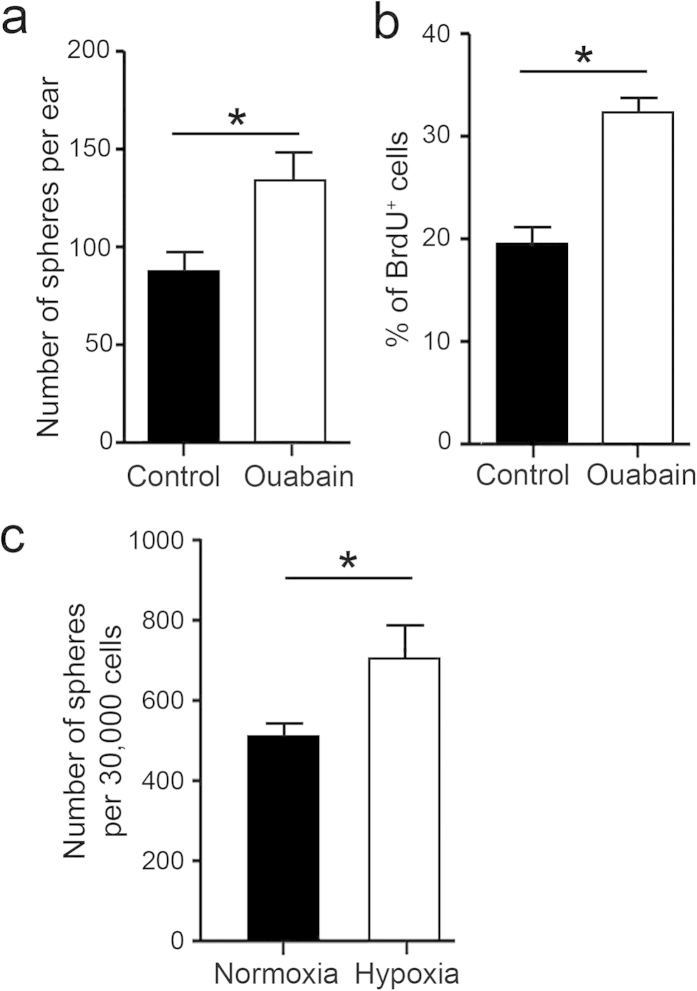
Acute injury (ouabain) and hypoxia enhance the formation of neurospheres from adult auditory nerves. (**a**) Ouabain exposure significantly increased the numbers of primary cochlear neurospheres generated from adult auditory nerves (n = 33 individual preparations for both ouabain-treated auditory nerves and the controls; *p < 0.05, Student’s unpaired t-test). (**b**) Ouabain exposure significantly increased the number of BrdU^+^ cells in neurospheres generated from adult auditory nerves (n = 25 for ouabain-treated nerves and n = 10 for the controls; *p < 0.05, Student’s unpaired t-test). (**c**) Hypoxia significantly enhanced neurosphere formation from adult auditory nerves (n = 3 per group; *p < 0.05, Student’s unpaired t-test).

Our previous study showed that acute injury to the auditory nerve induced by ouabain exposure significantly increased Sox2^+^ glial cells^22^. Here, our microarray analysis of ouabain-treated auditory nerves confirmed that Sox2 expression levels were significantly up-regulated at both 3 and 7 days following ouabain exposure (Supplementary Table 1). To test the hypothesis that acute injury to the adult auditory nerve increases the number of NSPs, we conducted a neurosphere formation assay on auditory nerves isolated from ouabain-exposed mice 3 days after treatment. As shown in Fig. 11a, the number of neurospheres generated from ouabain injured auditory nerves increased about 1.5-fold compared to controls. Analysis of proliferation using BrdU incorporation assays showed that neurospheres prepared from ouabain-injured nerves had significantly more BrdU^+^ cells than neurospheres prepared from controls (Fig. 11b). Under neural differentiation conditions, neurospheres generated from ouabain-injured ears were found to have Sox10^+^ and TuJ1^+^ cells at 29 ± 5% (n = 5) and 26 ± 2% (n = 4), respectively, which were levels that were roughly similar, albeit elevated, relative to neurospheres generated from normal adult ears (see Fig. 8g).

It has been shown that hypoxia within a certain range (e.g. 3 to 5% O_2_) is favorable for the maintenance of stem cell pluripotency^46^. To determine the effect of hypoxia on NSPs in the adult auditory nerve, we performed the neurosphere formation assay at the hypoxia level of 3% O_2_. As shown in Fig. 11c, hypoxic conditions significantly increased the yield of neurospheres from auditory nerves of adult CBA/CaJ mice compared to those that were cultured in a normoxic condition.

## Discussion

We have documented that a subpopulation of glial cells in the adult mouse cochlea acquire NSP properties after acute injury. Transcriptomic analysis of ouabain-exposed auditory nerves showed that acute injury caused up-regulation of numerous genes that are associated with neurogenesis and which are normally elevated during the early stages of postnatal auditory nerve development. Some of these genes, such as Sox2, Nestin, Vim, Gfap, and Ngfr (p75), are widely used as molecular markers of NSPs^32^,^33^,^34^. Neurosphere formation assays demonstrated that neurospheres could be generated from the entire adult mouse auditory nerve or a purified subpopulation of glial cells. Assays performed with a Sox2 eGFP mouse model determined that the neurosphere-forming cells in adult auditory nerve were a subpopulation of glial cells that express Sox2. Lastly, neurosphere formation was enhanced by injury with ouabain and in response to hypoxia.

An interesting finding of this study was that acute injury of adult SGNs caused up-regulation of numerous genes typically expressed in the early stages of postnatal nerve development. This reveals that the adult mouse auditory nerve is capable of reinitiating aspects of normal development in response to pathological conditions, such as acute nerve injury. Nestin and Vim are two molecular makers commonly used for the identification of NSPs in adult neural tissues^28^,^37^,^47^. These two genes exhibited the pattern of up-regulation in ouabain-injured auditory nerve and declining expression during postnatal auditory nerve maturation. In adult cerebral cortex, mature glial cells, which have limited capability to proliferate, do not express Nestin, Vim, tenascin C or Gfap^18^,^48^. However, after nerve injury, a subset of reactive glial cells expressed these proteins and presented typical NSP features of radial glial cells^18^,^49^.

The forkhead box transcription factor FoxD3 is a stemness gene that is required for maintenance of neural crest progenitors^36^,^50^. FoxD3 is also a key regulator of the neural lineage that arises from neural crest stem cells^35^,^36^. Our data revealed that FoxD3 was up-regulated in the auditory nerve with ouabain exposure and down-regulated with age during postnatal auditory nerve maturation. Additionally, FoxD3 was implicated as an important transcriptional regulator of the other 397 genes exhibiting this transcriptional profile (Z = 10.462), with FoxD3 TREs found in promoters of 160 of the genes. Analysis of these putative Foxd3 target genes detected functional associations with regulation of cell proliferation and nervous system development. Thus, FoxD3 may be a key factor in influencing how mature cochlear glial cells, within the appropriate microenvironment, switch to a dedifferentiated state and behave as multipotent NSPs.

Cellular and gene profiling evidence presented here support the hypothesis that acute auditory nerve injury stimulates quiescent glial cells to up-regulate genes that are highly expressed in NSPs, and thus, revert to a de-differentiated phenotype. This was also validated by neurosphere formation assays showing that a subgroup of Sox2^+^ glial cells is the source of NSPs in the adult auditory nerve. In the central nervous system, some astrocytes function as NSPs during adult neurogenesis^28^,^51^,^52^. A subpopulation of cells with morphological and immunophenotypic characteristics of astrocytes in the SVZ neurogenic zone expresses NSP markers. These cells contribute to olfactory neurogenesis *in vivo* and the formation of clonal, self-renewing multipotent neurospheres *in vitro*. In the injured brain, a subset of astrocytes up-regulates expression of Nestin and Gfap, proliferate, and becomes neurogenic after exposure to an enriched culture condition^49^,^53^. In addition, myelinating Schwann cells isolated from adult palatal ridges and sciatic nerves can be reprogrammed into multipotent NSPs under appropriate culture conditions^54^.

The transcription factor Sox2 is highly expressed in NSPs during adult neurogenesis^55^,^56^. Our previous study demonstrated that Sox2 up-regulation occurred in the glial cells of auditory nerves in adult mouse ears following type I SGN loss induced by ouabain exposure^22^. Here, global transcriptional analysis confirmed an up-regulation of Sox2 gene expression after ouabain exposure (Supplementary table 1). In addition, comparative analysis revealed that more than half of known Sox2 regulatory target genes^57^ are differentially expressed in ouabain-injured auditory nerves (Supplemental Fig. 2). These data, together with the results of our neurosphere formation assays in Sox2 eGFP transgenic mice showing that the Sox2^+^ glial cells are the resource of NSPs in the adult auditory nerve, demonstrate that Sox2 plays an important role in regulating the proliferation and de-differentiation of cochlear glial cells in adult cochlea after SGN acute injury.

A decrease in histone acetylation enacted by HDACs can result in condensed chromatin structure, abundant heterochromatin and transcriptional silencing^58^. Intensive expression of both HDAC1 and HDAC2 was detected in neural cells in the mouse central nervous system^59^,^60^. When NSPs differentiate into mature cells, expression of HDAC2 is up-regulated. Here, our data show that HDAC1 and HDAC2 were highly expressed in mature glial cells of the auditory nerves in control cochleas. However, shortly after ouabain exposure, production of these enzymes was down-regulated. These results suggest that down-regulation of both HDAC1 and HDAC2 in response to injury may contribute to the observed reduction in chromatin condensation, thereby influencing the injury-dependent de-differentiation of the glial cells in the adult cochlea.

Reactive gliosis (or glial scar formation) is a hallmark of brain injury. For years, the dense scarring and intense local inflammation with this condition were thought to inhibit regenerating axons from reaching their distal targets. However, recent studies have demonstrated that several injury conditions stimulate mature glial cells to acquire (or reactivate) NSP potential as part of reactive gliosis^18^,^19^,^49^,^61^. An important finding of this study was that acute injury in the auditory nerve significantly enhanced neurosphere formation in cultures of auditory nerve cells. These *in vitro* data support our *in vivo* gene expression results showing that acute injury in adult auditory nerve can initiate a program of gene expression resembling the early stages of postnatal auditory nerve development. Further functional experiments are necessary to determine whether the neurosphere cells generated from the auditory nerve can be fully differentiated into functional spiral ganglion neurons. The glial cells, including Schwann and satellite cells in the peripheral auditory nerve, are thought to develop from the neural crest (NC). It is still questionable if the NC-derived stem cell-like cells from adult cochlear nerve can fully differentiate into sensory neurons. It was reported that NC-derived stem cells fail to generate sensory neurons at the presence of BMP2 signaling or the absence of certain components of Wnt signaling^21^.

Another interesting result is that mild hypoxia augments neurosphere formation in cultures of cells from adult auditory nerve, suggesting that hypoxia plays a beneficial role in the survival or stemness of NSPs derived from the auditory nerve. Oxygen levels in the developing embryo are often low and this has been shown to influence development of organs such as the placenta and the vasculature^62^. Previous studies have demonstrated that hypoxia-inducible factor α1 facilitates signal transduction pathways involving Notch and bone morphogenetic proteins to promote cell proliferation and inhibit cell differentiation or apoptosis^63^. Mild hypoxia significantly enhanced self-renewal and multipotency of cultured NSPs and increased proliferation within the stem cell niche in an *in vivo* perinatal hypoxia/ischemia model^5^,^64^. Hypoxia has also been shown to induce de-differentiation of committed cells in other cell populations^65^. A more thorough understanding of the roles of hypoxia and its inducible factors in the regulation of auditory nerve-derived NSPs could lead to new methods for enhancing regeneration or self-repair of injured auditory nerves.

Although our study found that the glial cells (at least a subset of them) in adult auditory nerve have several characteristics of NSPs, no evidence of auditory nerve regeneration has been identified in the ouabain mouse model or other *in vivo* animal models of sensorineural hearing loss. To better understand this limitation, we examined the expression of Notch signaling-associated genes in response to injury as compared to the response seen in auditory nerve derived neurospheres, which represented a more clearly regenerative state (Supplementary Fig. 3). While some Notch pathway genes showed injury response patterns that resembled the regenerative state (e.g., upregulation of Dll1 and Hes1 under both conditions; Supplementary Fig. 3b), other Notch pathway genes showed patterns that opposed the regenerative state (e.g., opposing regulation of Jagged 1 and Hey 1 under the two conditions; Supplementary Fig. 3a). These results suggest that certain components of Notch signaling fail to follow a full regenerative response. Another possible factor contributing to the inhibition of auditory nerve regeneration is the direct effect of ouabain exposure on the glial cells. It was reported that ouabain exposure alters inflammatory reactivity and influences specific types of glial cells in *in vitro* conditions^66^,^67^.

Using a mouse model of auditory nerve injury, combined with multidisciplinary *in vivo* and *in vitro* approaches, we demonstrated that acute injury stimulates quiescent glial cells in the adult auditory nerve to up-regulate genes that are highly expressed in developing auditory nerve and revert to a de-differentiated phenotype. What is striking about our findings is the remarkable plasticity of the glial cells in the adult mouse auditory nerve. Since the SGNs have a limited capacity to regenerate, their death results in permanent sensorineural hearing loss. Cochlear glial cells represent potential for interventions that attempt to regenerate or repair SGNs. A recent study showed that modifying expression of a single transcription factor Sox2 can convert glial cells into proliferative neuroblasts, giving rise to functional neurons *in vivo* when supplied with BDNF and Noggin or treated with a histone deacetylase inhibitor^68^. Further studies of the cell-intrinsic properties and environmental cues that control proliferation and neural differentiation of NSPs will be necessary for understanding the capability to replace lost neural tissue and restore auditory function.

## Methods

### Animals

Adult CBA/CaJ and Sox2 transgenic mice were bred in-house in a low-noise environment at the Animal Research Facility of the Medical University of South Carolina (MUSC). The original adult CBA/CaJ breeding pairs were purchased from The Jackson Laboratory (Bar Harbor, ME). Sox2 eGFP transgenic mice were kindly provided by Dr. Fred Gage^45^. All mice received food and water *ad libitum* and were maintained on a 12-hr light/dark cycle. Adult mice aged 6–12 weeks and postnatal (P) developing mice at ages P0, 3, 7, 10, 14, and 21 of both genders were used in the study. Prior to data acquisition, adult mice were examined for signs of external ear canal and middle ear obstruction. Mice with any symptom of middle ear infection were excluded from the study. All vertebrate animal experimentation was performed in accordance with institutional and federal guidelines and regulations. Vertebrate animal experimental protocols were reviewed and were approved by the Institutional Animal Care and Use Committee of Medical University of South Carolina.

### Physiological procedures

Adult mice before and after ouabain treatment were processed for auditory physiological measurements. Auditory brainstem responses (ABRs) were measured as previously described^22^. Mice were anesthetized by an intraperitoneal injection of xylazine (20 mg/kg) and ketamine (100 mg/kg) and placed in a sound-isolation room. The acoustic stimuli were generated using Tucker Davis Technologies equipment System III (Tucker-Davis Technologies, Gainsville, FL, USA) and a SigGen software package (Version 4.4.1). Calibration was completed using a Knowles microphone in a probe tube clipped to the pinna. The signals were delivered into the animal ear canal through a 10 mm long (3–5 mm diameter) plastic tube. ABRs were evoked at half octave frequencies from 4 to 45 kHz with 5 ms duration tone pips with cos^2^ rise/fall times of 0.5 ms delivered at 31 times/s. Sound levels were reduced in 5 dB steps from 90 dB SPL to 10 dB SPL below thresholds. Physiological results of each mouse were analyzed for individual frequencies, and then averaged for each of these frequencies from 4.0 to 45 kHz.

### Ouabain exposure

Surgical procedures were modified from previous studies^22^. Briefly, mice were anesthetized with xylazine (10 mg/kg, i.p.) and ketamine (100 mg/kg, i.p.). Survival surgery was performed under sterile conditions. Buprenorphine (0.1 mg/kg, i.p., once 30 min before the surgery) was administered to minimize any surgical discomfort. Sterile procedures were used to open the bulla and deliver ~10 μl of 3 mM ouabain (Sigma-Aldrich, O3152) solution to the round window niche. The total time of ouabain exposure was approximately 60 min; every 10 min the ouabain solution was wicked away with filter paper wicks prepared preoperatively and a fresh solution was applied. The right ear was the operative ear while the left ear served as a control. Following treatment with ouabain, adult mice were allowed to recover for either 3 or 7 days. Ouabain treated mice exhibiting ABR wave I threshold shifts of less than 30 dB were excluded from subsequent experimentation.

### Cochlear nerve tissue collection and total RNA isolation

Adult mice treated with ouabain and postnatal mice were euthanized and the cochleas were collected. Microdissection was performed to remove the outer bony cochlear shell, cochlear lateral wall, and the majority of sensory epithelium, thereby preserving the modiolus portion of the cochlea where the auditory nerve is located. For ouabain exposed mice, control (left ear) and treatment (right ear) cochleas were prepared separately; tissues from two control or treatment preparations were pooled for individual samples. For postnatal mice, the left and right ear cochlea preparations from a single mouse were pooled for individual samples. Total RNA was purified from cochlea preparations using the miRNeasy Mini Kit (Qiagen Inc, Germantown, MD) according to the manufacturer’s instructions.

### Microarray hybridization and data analysis

Procedures for probe preparation, hybridization, and microarray data processing were modified from those described previously^69^. Total RNA preparations were assessed for quality using the 2100 Bioanalyzer (Agilent Technologies, Santa Clara, CA). Ouabain study samples (n = 3) and postnatal samples (n = 2) of high quality total RNA were selected for use. Total RNA (ouabain study, 50 ng; postnatal study, 100 ng; neurosphere study, 100 ng) was converted into biotin-labeled cRNA using the 3′ IVT Express Kit (Affymetrix, Santa Clara, CA) according to the manufacturer’s instructions. Hybridization of biotin-labeled cRNA to Mouse 430 2.0 GeneChips (Affymetrix) and post hybridization washing, staining, and fluorescence scanning were conducted using Affymetrix instrumentation following manufacturer recommendations. Raw hybridization data (CEL files) for each study were normalized by both Robust Multi-array Average (RMA)^70^ and Microarray Suite 5.0 algorithms using Expression Console software (Affymetrix). Comparisons were performed using dChip software^71^. For the ouabain treatment study, genes differentially expressed were identified as those with absolute fold change >1.5 and *p* < 0.05 (Student’s unpaired t-test) for ouabain treatment versus control at either 3 days or 7 days after ouabain exposure (identifying 6915 differentially expressed genes). For the postnatal development study, differential expression was defined as absolute fold change >1.5 and *p* < 0.05 (Student’s unpaired t-test) for P3, P7 or P10 versus P0 (identifying 6023 genes). For the neurosphere study, differential expression of stemness genes^43^ was defined as *p* < 0.05 (Student’s unpaired t-test) for neurosphere cultures versus auditory nerve tissue (identifying 346 genes). False discovery rate (FDR) for comparative analysis involving all microarray content was estimated with dChip software using the iterative randomization method, which calculates FDR as the median number of genes discovered by iterative comparisons involving randomized sample group assignments divided by the genes discovered using correct sample assignments. Calculations based on 50 iterations estimated FDR as <5% for all comparisons. Raw microarray data files associated with these studies were deposited in NCBI Gene Expression Omnibus under the series accession GSE59417.

### Detection of enriched transcription factor binding sites

Hierarchical clustering of the 636 genes showing the pattern of induction following ouabain injury and reduction during postnatal development revealed two predominant sets that differed in their response to ouabain injury. One subset of 398 genes showed sustained up-regulation at both 3 days and 7 days following ouabain treatment, while the second set of 186 genes was only transiently up-regulated at 3 days. To address the hypothesis that sustained up-regulation was likely to be representative of a regenerative response following SGN degeneration, the subset of 398 genes was chosen for analysis of transcription factors implicated in regulating neurogenesis and neural development events. The 398 genes were analyzed for enrichment of transcriptional response elements (TREs) in their promoters using the oPOSSUM 3.0 tool^67^ configured for single site analysis of promoter flanking sequences extending from −2000 to +2000 with respect to gene transcription start sites. Significant TREs were evaluated based on Z-scores.

### Quantitative RT-PCR

Reverse transcription of total RNA was performed with the QuantiTect Reverse Transcription Kit (Qiagen, Germantown, MD) according to the manufacturer’s protocol. Briefly, genomic DNA was eliminated from RNA samples by incubation with gDNA Wipeout Buffer for 2 min at 42 °C. Reverse transcription was performed with 50 ng of total RNA and Quantiscript Reverse Transcriptase in 20 μl reaction volumes incubated at 42 °C for 15 min and 95 °C for 3 min. The following gene-specific amplification reagents were purchased from Qiagen : 18S (QT02448075), Ngfr (QT01047004), Nestin (QT01051547), Gdnf (QT00115290), Sox2 (QT00249347), Nedd4l (QT01069355), Gfap (QT00101143), Ednrb (QT00139384), Foxd3 (QT00252056), Vim (QT00159670), Dcx (QT02521155), Fgf10 (QT00164157), Erbb3 (QT01168167), Fabp7 (QT00109865), Gap43 (QT00101955), Pax6 (QT01052786), BDNF (QT00097118), Notch 1 (QT00156982), and Shh (QT00122479). Forward and reverse primers for Calb were purchased from Invitrogen (10336022). The Quantitative PCR reactions were performed with the QuantiFast SYBR Green PCR Kit (Qiagen) using 1 μl of cDNA and a LightCycler 480 (Roche Diagnostics, Indiana, USA). Negative controls included reactions lacking cDNA template and reverse transcriptase. 18s rRNA was used as a reference gene for normalization. All reactions were performed in technical triplicate. Cycling parameters for PCR were: 50 °C for 2 min, activation at 95 °C for 5 min, and 40 cycles of 95 °C for 10 sec and 60 °C for 30 sec.

### Immunohistochemistry

After physiological recording, cochleas were fixed with 4% paraformaldehyde solution for 1–2 hours at RT, decalcified with 0.12 M ethylenediamine tetraacetic acid (EDTA) at RT (stirring), and dehydrated. The tissues were then embedded in Tissue-Tek OCT compound, and sectioned at 10 μm thickness. For neurosphere analysis, neurospheres were collected, washed with phosphate buffered saline (PBS), and fixed with 4% paraformaldehyde for 20 min at RT. After fixation, neurospheres were embedded in 4% gelatin. Sections were immersed in blocking solution for 20 min and then incubated overnight at 4 °C with a primary antibody diluted in 0.2% BSA. The primary antibodies used in this study are listed in Supplementary Table 6. Secondary antibodies were biotinylated and binding was detected by labeling with fluorescein (FITC)-conjugated avidin D or Texas red-conjugated avidin D (Vector Labs, Burlingame, CA). Nuclei were counterstained with propidium iodide (PI), bisbenzimide or DAPI. Sections were examined on a Zeiss LSM5 Pascal confocal microscope or Olympus Fluoview FV1000 confocal microscope. FITC and Texas red (or PI) signals were detected by excitation with the 488 nm and 543 nm lines, respectively. Images were scanned at scales of 0.29 μm (x) x 0.29 μm (y) and a stack size of 146.2 μm (x) x 146.2 μm (y) with a Plan-Apochromat 63×/1.4 Oil DIC objective (Carl Zeiss, Germany). Captured images were processed using Zeiss LSM Image Browser Version 3,2,0,70 (Carl Zeiss Inc., Jena, Germany) and Adobe Photoshop CS.

### Transmission electron microscopy

Anesthetized animals were perfused via cardiac catheter with 10 ml of normal saline containing 0.1% sodium nitrite followed by 15 ml of a mixture of 4% paraformaldehyde and 2% glutaraldehyde in 0.1 M phosphate buffer, pH 7.4. After removing the stapes and opening the oval and round windows, 0.5 ml of the same fixative described above was perfused gently into the scala vestibuli through the oval window. Inner ears were dissected free and immersed in fixative overnight at 4 °C. Decalcification was completed by immersion in 40 ml of 120 mM solution of EDTA, pH 7.0, with gentle stirring at room temperature for 2–3 days with daily changes of the EDTA solution. For neurosphere analysis, the neurospheres were centrifuged and fixed with the same mixture for 2 hours at RT. Cochlear tissues and neurospheres were postfixed with 1% osmium tetroxide-1.5% ferrocyanide for 2 hours in the dark, then dehydrated and embedded in Epon LX 112 resin. Semi-thin sections approximately 1 μm thick were cut and stained with toluidine blue. Ultrathin sections (70 nm thick) were stained with uranyl acetate and lead citrate and examined by electron microscopy.

### Heterochromatin Density Analysis

Quantification of the heterochromatin density of glial cells before and after ouabain treatment was performed by processing electron microscopy images with AxioVision 4.8 (Carl Zeiss, Inc.) software. Nucleus and heterochromatin values were measured using the outline and measurement tool in the software. Heterochromatin pixel percentages were calculated using the following equation: heterochromatin region pixel values/whole nucleus region pixel values × 100. Twelve measuring regions from control mice, five regions from 1 day post treatment mice, and five regions from 3 day post treatment mice were examined. All the measured regions were randomly selected on the cochlear modiolus sections. Mean heterochromatin density percentages were compared using unpaired Student’s t-tests and differences between means were considered statistically significant when *p* < 0.05.

### Neurosphere assay

Animals were anesthetized and decapitated under a sterilization condition. Brain tissues were removed and the temporal bones transferred to a culture dish containing 5–10 ml HBSS (Hanks balanced salt solution, Sigma) and washed twice with HBSS. Auditory nerves were removed from the temporal bones and incubated with 0.25% trypsin for 5 min at 37 °C. After trypsin treatment, auditory nerves were incubated with DNase I (final concentration of 5 mg/ml) for 5 min at 37 °C. Finally, 10% fetal bovine serum (FBS) containing DMEM/F12 medium was added to stop trypsin reaction. Tissues were triturated with pipet tips and centrifuged at 1000 rpm for 5 min. The pellet was resuspended in DMEM/F12 medium with 10% FBS and B27 supplements and filtered through a 70 μm cell strainer. Cells were counted, plated on poly-L-ornithine and fibronectin coated plates, and grown to full confluency (5–7 days). Media was then removed and replaced with serum free proliferation medium (NeuroCult^TM^ proliferation supplement, Stem Cell Technologies) containing 20 ng/ml rh EGF, 10 ng/ml FGF and 2 μg/ml heparin.

The procedure for the purification of cochlear glial cells using immune-magnetic sorting was modified from previously described^44^ and from the instructions provided by the manufacturer (Miltenyi Biotech, Germany). Cells were re-suspended and washed using PBS containing 1% bovine serum albumin (PB1), then incubated with rabbit anti-P75 NGF receptor (Millipore-Chemicin, Billsrica, MA, USA) cells at 4 °C for 20 min. After washing twice with PB1, cells were re-suspended in PBS containing 0.5% bovine serum albumin and 2 mM EDTA (PBE), then were incubated with MACS micro-beads coupled to anti-rabbit IgG microbeads (Miltenyi Biotec, USA) at 4 °C for 15 min. After incubation, cells were washed twice and re-suspended with PBE, then added to the top of a prewashed magnetic column. The flow-through cells were washed through with three applications. The column was removed from the magnet and 1 ml of PBE was gently pushed through with a plunger. The cell sorting procedure was performed twice to enhance the enrichment of the P75^+^ glial cell population. We also purified the Thy-1.2^+^ fibroblasts as a negative control (non-glial cell enriched fraction) using the same MACS protocol described above. The primary and second antibody for immunemagnetic sorting were rat anti Thy1.2 (AbD Serotec, US) and anti-rat IgG microbeads (Miltenyi Biotec, USA), respectively.

For culture with hypoxic condition, hypoxic conditions (O_2_ concentration: 3%) were maintained in a humidified 5% CO_2_ multi-gas incubator by continuous flushing with 92% N_2_ gas. For the BrdU assay, neurospheres were incubated with 9.8 μM BrdU (Sigma) at 37 °C overnight as previously described^21^,^39^. Neurospheres were transferred to 4-well Lab-Tek chamber slides (Fisher Scientific) coated with poly-L-ornithine and fibronectin (Sigma) and incubated for 5 days with neural differentiation medium (NeuroCult differentiation supplements, Stem Cell Technologies) containing 1 ng/ml of leukemia inhibitory factor (Chemicon) at 37 °C. Medium was changed every 3 days. Cultures were maintained for up to 14 days in culture before fixation for immunohistochemistry. The fibroblast L Cell line (ATCC^@^ CRL-2648^TM^, American Type Culture Collection, Manassas, VA) was used as a negative control for the neural differentiation assay on neurosphere cells.

### Data processing and statistical analyses

Unless otherwise specified, all data in the figures are presented as mean ± standard error of the mean (SEM). Data for the density of Sox10^+^ and BrdU^+^ cells, density of Sox10^+^/BrdU^+^ cells, heterochromatin percentages, density of HADC^+^, Gfap^+^, Sox10^+^ and TuJ1^+^ cells, number of neurospheres, as well as quantification of mRNA expression levels by quantitative PCR for Ngfr, Nestin, Gdnf, Sox2, Nedd4l, Gfap, Ednrb, FoxD3, Vim, Fgf10, Dcx, Calb, and Shh were analyzed by one- and two-way ANOVA or two tailed, unpaired Student’s *t* test using statistics software (SPSS, Chicago, IL). A value of *p* < 0.05 was considered to be statistically significant.

## Additional Information

**How to cite this article**: Lang, H. *et al.* Neural stem/progenitor cell properties of glial cells in the adult mouse auditory nerve. *Sci. Rep.* **5**, 13383; doi: 10.1038/srep13383 (2015).

## Supplementary Material

Supplementary Information

Supplementary Table 1

Supplementary Table 2

Supplementary Table 3

Supplementary Table 4

Supplementary Table 5

## Figures and Tables

**Table 1:** Transcriptional response elements implicated in the mouse auditory nerve as regulating induction following injury and down-regulation during postnatal maturation.

**Transcriptional response element (TRE)**^1^	**Z-score**^2^	**Target gene hits (of 329 analyzed)**^3^
SP1	21.6	233
Klf4	20.7	240
MZF1_5–13	17.0	226
MZF1_1–4	11.5	270
Foxd3	10.5	160
NR2F1	10.3	63
Nobox	10.2	208
Sox5	10.0	201
Egr1	9.1	115
Pax4	8.9	3

^1^Significant TREs were calculated using the oPOSSUM tool^72^,^73^ configured to analyze promoter sequences extending from 2000 base pairs upstream to 2000 base pairs downstream of gene transcription start sites.

^2^Z-score is a measure of enrichment of the TRE among the analyzed group compared to a pre-computed background set.

^3^398 genes were chosen for analysis based on microarray expression patterns in mouse adult auditory nerve of 1) up-regulation at both 3 days and 7 days after ouabain exposure, and 2) high expression at P0, but down-regulation during postnatal maturation. 329 genes remained after excluding redundant and unknown genes.
