# Supplementary material for: Neural stem/progenitor cell properties of glial cells in the adult mouse auditory nerve
Source: Sci Rep. 2015 Aug 26;5:13383. doi: 10.1038/srep13383 (PMC4549618; doi:10.1038/srep13383)
Supplement: Supplementary Information [file srep13383-s1.pdf]

Supplementary Information

Neural stem/progenitor cell properties of glial cells in the adult mouse auditory nerve

Author names and affiliations: Hainan Lang[1], Yazhi Xing[1], LaShardai N Brown[1], Devadoss J Samuvel[1], Clarisse H Panganiban[1], Sundaravadivel Balasubramanian[3], Luke T Havens[1], Michael Wegner[4], Edward L Krug[2], Jeremy L Barth[2]

[1]Department of Pathology and Laboratory Medicine, Medical University of South Carolina, Charleston, South Carolina 29425, United States; [2]Department of Regenerative Medicine and Cell Biology, Medical University of South Carolina, Charleston, South Carolina 29425, United States; [3]Department of Radiation Oncology, Medical University of South Carolina, Charleston, South Carolina 29425, United States; [4]Institute of Biochemistry, Friedrich-Alexander-University Erlangen-Nürnberg, Erlangen 91054, Germany

Supplementary Figure 1: A heatmap of the 636 genes that were associated with ouabain injury and early auditory nerve development. Expression is also shown for early vestibular ganglion (VG) development based on microarray data from a previous report [16]. A large portion of the 636 genes showed elevated expression in VG at embryonic day 16 and P0.

Supplementary Figure 2: Sox2-dependent genes are differentially expressed in the injured auditory nerve 3 days after ouabain exposure. Sox2-dependent genes were identified in NSCs by independent microarray and chromatin immunoprecipitation-ChIP assays [43]. 242 of 418 are established Sox2-activated genes).

Supplementary Figure 3: Differential expression analysis of Notch signaling associated genes in 1) the injured adult auditory nerve 7 days after ouabain exposure versus controls; and 2) adult auditory nerve cell culture (with a standard tissue culture protocol with serum) (ACC) versus adult auditory nerve-derived neurosphere cultures (ANC). Significant difference is defined as $p<0.05$, student's unpaired t-test (n=3 per group).

Supplementary Table 1: 6915 genes are differentially expressed between controls and ouabain treatment at either 3 days or 7 days after ouabain exposure.

*Note that **Supplementary Table 1** is a larger supplementary dataset and is submitted separately in .xls format.

Supplementary Table 2: 6023 genes are differentially expressed in the auditory nerve during postnatal development.

*Note that **Supplementary Table 2** is a larger supplementary dataset and is submitted separately in .xls format.

Supplementary Table 3: 636 genes showed significant up-regulation in adult auditory nerve by ouabain injury and also down-regulation with age in postnatal developing nerve.

*Note that **Supplementary Table 3** is a larger supplementary dataset and is submitted separately in .xls format.

Supplementary Table 4: 636 genes showed significant up-regulation in adult auditory nerve by acute injury and down-regulation with age in postnatal developing nerve also showed a similar expression pattern in developmental VG tissues based on a previous study [16].

*Note that **Supplementary Table 4** is a larger supplementary dataset and is submitted separately in .xls format.

Supplementary Table 5: Differential expression analysis of stemness genes in adult auditory nerve tissue (ANT) versus adult auditory nerve-derived neurosphere cultures (ANC). Significant difference is defined as $p<0.05$, student's unpaired t-test.

*Note that **Supplementary Table 5** is a larger supplementary dataset and is submitted separately in .xls format.

Supplementary Table 6: Characterization of antibodies used in the immunohistochemical study.

Supplementary Figure 1

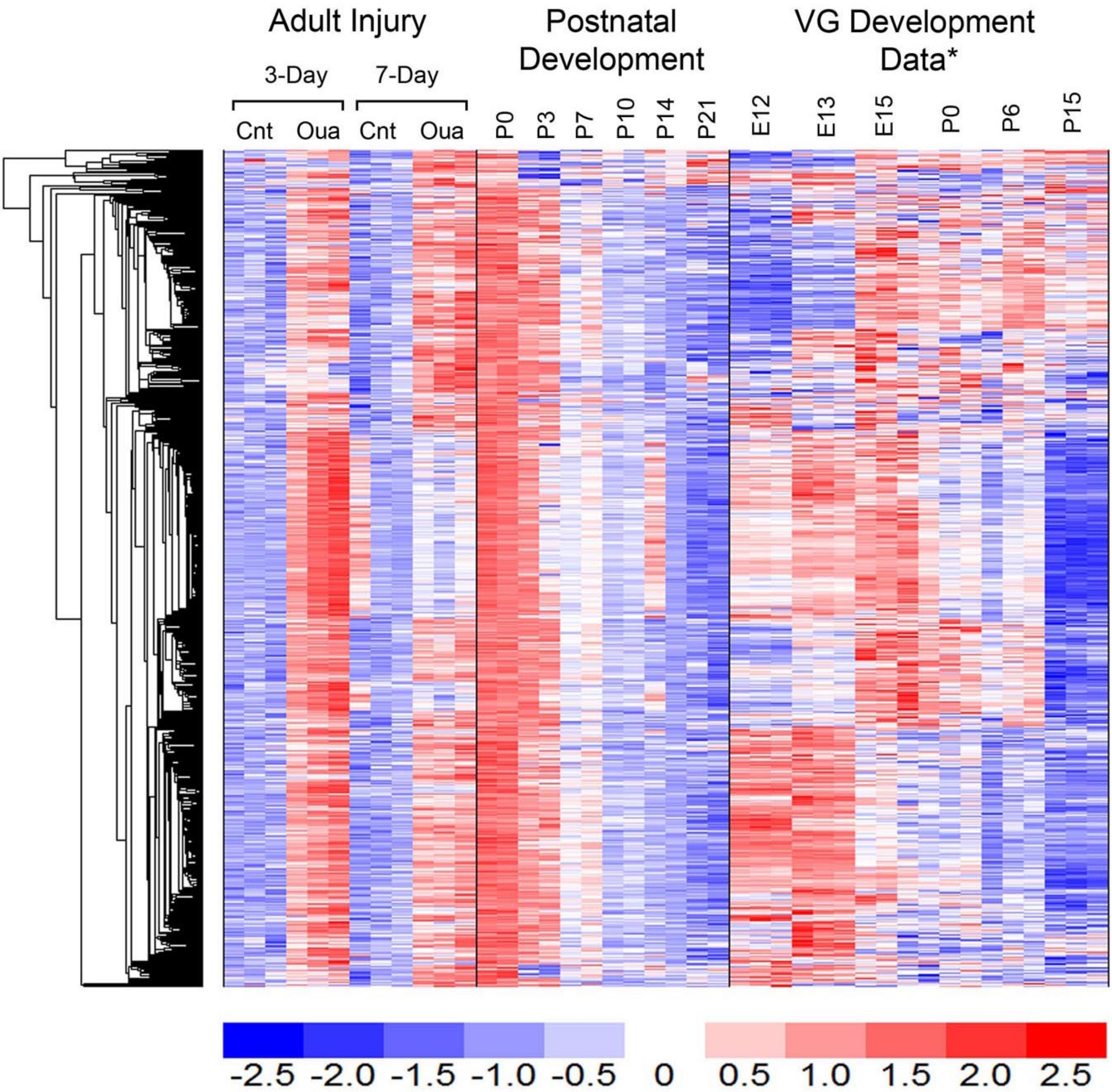

Supplementary Figure 2

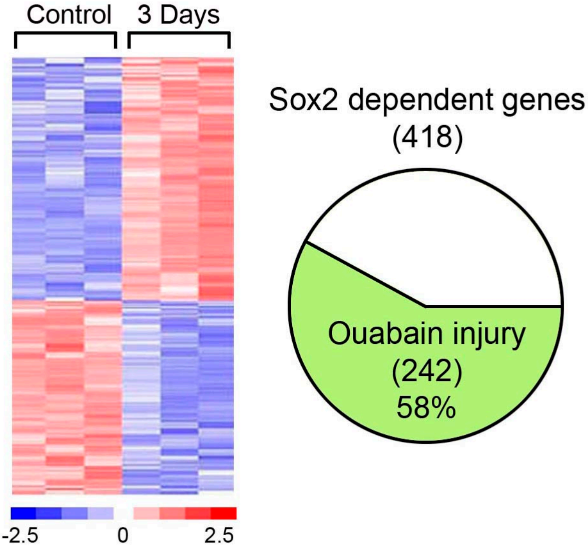

Supplementay Figure 3

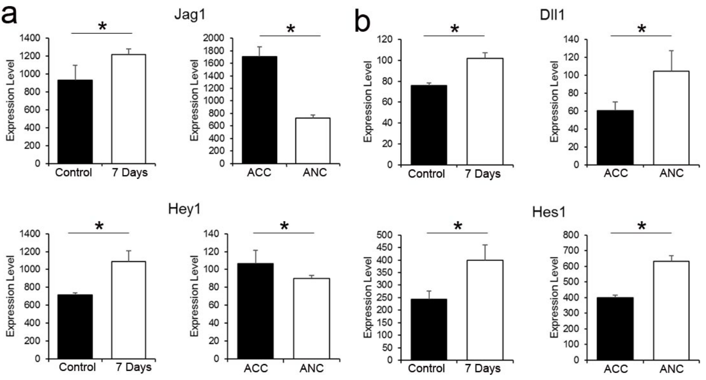

Supplementary Table 6. Characterization of antibodies used in the immunohistochemical and cell sorting assay.

Antibody	Immunogen	Species	Catalog No	Supplier	Dilution
BrdU	Antibody raised against BrdU incorporated into DNA or coupled to a protein carrier	Mouse	B2531	Sigma	1: 500
GFP	Antibody raised against rabbit IgG fraction.	Rabbit	A11122	Molecular probes	1: 100
GFAP	Purified GFAP from porcine spinal cord	Mouse	MAB360	Chemicon	1: 300
Fibroblast	Murine thymic reticulum	Rat	BM4018	Acris	1: 400
β Tubulin III	Synthetic peptide corresponding to the carboxyl-terminal sequence of human β-tubulin isotype III conjugated to BSA.	Mouse	T8660	Sigma	1: 300
S100 β	Antibody raised against purified bovine brain S100 β	Mouse	S2532	Sigma	1: 350
β Tubulin III	Antibody raised against the microtubules from rat brain.	Rabbit	MRB-435P	Covance	1: 300
Sox2	Antibody raised against a peptide mapping near the C-terminus of human Sox2	Goat	sc-17320	Santa Cruz	1: 100
Sox10	Antibody raised against a peptide mapping at the N-terminus of human Sox-10	Goat	sc-17342	Santa Cruz	1: 300

Sox10	Polyclonal antibody raised against a purified bacterially expressed protein consisting of amino acids 181-233 and 308-400 of rat Sox10	Guinea pig		A gift from Dr. Wegner	1:500
Nestin	Antibody purified from embryonic rat spinal cord.	Mouse	MAB353	Millipore	1:250
NGF receptor P75	GST-tagged NGF receptor P75 corresponding to the cell membrane.	Rabbit	AB1554	Millipore	1:10
α smooth muscle actin	N-terminal synthetic decapeptide of α-smooth muscle actin.	Mouse	A2547	Sigma	1:400
HDAC1	A synthetic peptide frm C-terminal of human HDAC1 protein.	Rabbit	AP15592PU	Acris	1:500
HDAC2	Synthetic peptide derived from the C-terminal region of mouse HDAC2	Rabbit	51-5100	Invitrogen	1:250
Neurofilament, Heavy	Purified bovine NF-H	Chicken	AB5539	Millopore	1:200
Thy-1.2	EL-4 cells	Rat	MCA1474	AbD Serotec	1:10
